# Functional polymorphisms in circadian positive feedback loop genes predict postsurgical prognosis of gastric cancer

**DOI:** 10.1002/cam4.2050

**Published:** 2019-03-07

**Authors:** Yibing Chen, Dandan Wang, Yucen Song, Xiaofei Zhang, Zhihui Jiao, Juqin Dong, Lin Lü, Zhengzhi Zou, Wei Du, Falin Qu

**Affiliations:** ^1^ Genetic and Prenatal Diagnosis Center First Affiliated Hospital Zhengzhou University Zhengzhou China; ^2^ Shandong Medicinal Biotechnology Centre, Key Laboratory for Rare & Uncommon Diseases of Shandong Province Back and Neck Pain Hospital, Shandong Academy of Medical Sciences Jinan China; ^3^ Department of Medical Oncology First Affiliated Hospital Zhengzhou University Zhengzhou China; ^4^ Cell‐Gene Therapy Translational Medicine Research Center The Third Affiliated Hospital of Sun Yat‐sen University Guangzhou China; ^5^ Department of Medical Oncology Guangzhou First People's Hospital Guangzhou Medical University Guangzhou China; ^6^ The Second Affiliated Hospital South China University of Technology Guangzhou China; ^7^ MOE Key Laboratory of Laser Life Science and Institute of Laser Life Science College of Biophotonics South China Normal University Guangzhou China; ^8^ Department of Neurosurgery First Affiliated Hospital Zhengzhou University Zhengzhou China; ^9^ Department of General Surgery Tangdu Hospital Fourth Military Medical University Xi'an China; ^10^ 93926 Hospital of the PLA Hetian China

**Keywords:** circadian gene, gastric cancer, prognosis, single‐nucleotide polymorphism

## Abstract

**Background:**

Circadian positive feedback loop (CPFL) genes (*CLOCK*,* BAML1*, and *NPAS2*) have been implicated in cancer initiation and progression. The purpose of this study was to explore the effects of single‐nucleotide polymorphisms (SNPs) in CPFL genes on prognosis of gastric cancer (GC) patients.

**Methods:**

Nine functional SNPs from the three CPFL genes were genotyped in a cohort of 704 GC patients undergoing resection. Multivariate Cox regression model and Kaplan‐Meier curve were used for prognosis analysis.

**Results:**

Among the nine SNPs, rs11133399 in *CLOCK*, rs1044432 and rs2279284 in *BAML1* were significantly associated with GC overall survival and recurrence‐free survival. The unfavorable genotypes of these SNPs showed a cumulative effect on GC prognosis. Multivariate assessment model indicated that these SNPs, in conjunction with clinical variables, enhanced the power to predict GC prognosis. In addition, survival tree analysis revealed the genotype of rs11133399 as a primary risk factor contributing to the prognosis of GC patients. Functional assays showed that the G allele in rs11133399 significantly enhanced luciferase reporter activity than A allele. Immunohistochemical analysis further demonstrated that the genotype of rs11133399 was significantly associated with the expression level of *CLOCK* in GC tissues, suggesting that this SNP might affect the prognosis of GC through its influence on the expression of *CLOCK* gene.

**Conclusions:**

Our data indicate that SNPs in CPFL genes might contribute to the clinical outcome of GC through their impact on gene expression. Further studies are needed to elucidate its underlying molecular mechanisms.

## 1 INTRODUCTION

Gastric cancer (GC) is one of the most common malignancies worldwide, with more than 950 000 newly diagnosed cases and 720 000 deaths each year.^1^ Despite great advances in early detection and treatment of GC in the past two decades, GC survival rate has shown only marginal increase due to the complexity and heterogeneity of molecular mechanisms during tumor progression, especially invasion, recurrence, and metastasis. Therefore, it is urgent to develop molecular biomarkers to elucidate the molecular mechanism for improving diagnosis and treatment of GC.

Circadian rhythms are endogenous biological oscillations with a period near 24 hours driven by the autonomous circadian clock.^2^ The molecular mechanisms of circadian clock are based on the positive/negative feedback loops generated by core circadian clock genes. Among them, *CLOCK*,* NPAS2*, and *BMAL1* form a circadian positive feedback loop (CPFL). Over the past few decades, accumulating evidence has suggested that circadian clock disruption is a contributory factor in tumor initiation and progression. Epidemiological studies have revealed that night shift work significantly increases the risk of breast, prostate, and rectal cancer (colorectal cancer [CRC]),^3^, ^4^, ^5^ indicating a possible functional link between molecular clock machinery and carcinogenesis. Subsequently, aberrant expression of circadian genes observed in many cancers further strengthens this appealing kinship.^2^ Previous studies have also demonstrated that abnormal expression of circadian clock genes is associated with the prognosis of cancer patients.^6^ These findings highlight the vital role of circadian clock genes in tumorigenesis and cancer developing.

Single‐nucleotide polymorphism (SNP) is the most common genetic variant in human genome, which is considered as a stable biomarker of genetic background to predict the risk, treatment response, and progression of human diseases.^7^ Previous studies have demonstrated that several SNPs are associated with the development and progression of GC.^8^, ^9^ Moreover, emerging evidence has shown that SNPs in circadian pathway are involved in cancer predisposition.^10^, ^11^ For example, several studies have suggested that rs2305160 in *NPAS2* gene contributes to the susceptibility of breast cancer.^12^ Further evidence has indicated that SNPs in circadian negative feedback loop (CNFL) genes are closely related to prostate cancer risk and prognosis of hepatocellular carcinoma (HCC).^13^, ^14^ A recent study has indicated the potential association of circadian gene polymorphisms with the prognosis of GC.^15^ Our previous findings have also suggested that functional SNPs in CNFL genes are significantly associated with prognosis of GC patients.^16^ The ensemble of these studies depicts a scenario that circadian gene polymorphisms could affect the initiation and development of cancer. However, due to the limited size of population, the association between functional SNPs in CPFL genes and GC prognosis needs to be validated in larger populations.

In the present study, we assessed the effects of nine functional SNPs in CPFL genes on the prognosis of 704 Chinese GC patients after surgery. Additionally, the effects of an identified relevant SNP rs11133399 on the transcriptional activity and expression of *CLOCK* gene were further evaluated by *in vitro* functional assays.

## 2 METHODS AND MATERIALS

### 2.1 Patients

A total of 704 Han Chinese GC patients who received surgical treatment at the Department of General Surgery, Tangdu Hospital, Fourth Military Medical University (Xi'an, China) from January 2008 to June 2013 were enrolled in this study. Patients who met all the following criteria were included: (a) newly diagnosed and histologically confirmed with primary gastric adenocarcinoma; (b) no previous history of other cancers; (c) receiving curative surgery, but without any preoperative anticancer treatment; (d) no blood transfusion within 3 months; (e) with complete epidemiological data, clinical information, and follow‐up data. Tumor staging was determined according to the 8th edition tumor‐node‐metastasis (TNM) Classification of the Union for International Cancer Control and American Joint Committee on Cancer.^17^ Lauren's criteria were used to classify the tumors into intestinal‐type or diffuse‐type GC.^18^ Clinical information was obtained through medical record and follow‐up review was performed by clinical specialist through telephone calling, outpatient review, or medical records at 6‐month intervals. The latest follow‐up data were obtained in February 2017. Overall survival (OS) was defined as the interval from initial surgery to death of any cause. Recurrence‐free survival (RFS) was defined as the interval from initial surgery to local recurrence or distant metastasis, whichever occurs first. This study was approved by the Ethic Committees of Fourth Military Medical University and Zhengzhou University, and written informed consent was obtained from all participants. All study procedures were performed in accordance with the Declaration of Helsinki, 1964 and later versions.

### 2.2 DNA extraction, SNP selection, and genotyping

Leukocyte DNA was extracted from 5 mL venous blood of patients using the EZNA blood Midi Kit (Omega Bio‐Tek, Norcross, GA, USA) according to the manufacturer's recommendation. The candidate functional SNPs in CPFL genes were selected using a set of web‐based SNP selection tools (http://snpinfo.niehs.nih.gov/snpfunc.htm) as previously described.^16^ Finally, nine potential functional SNPs, including three in *CLOCK* gene, two in *BAML1* gene, and four in *NPAS2* gene, were selected for genotyping with Sequenom iPLEX genotyping platform (Sequenom Inc., San Diego, CA, USA) according to the manufacturer's protocol. Strictly quality controls were performed in each assay during genotyping. SNPs with a call rate >98% were included for further analysis.

### 2.3 Cell culture

Human GC cell lines, SGC‐7901 and AGS, were obtained from the Cell Bank of the Chinese Academy of Sciences (Shanghai, China) and cultured as previously described.^19^ All cells were *Mycoplasma*‐free and authenticated by short tandem repeat DNA profiling analysis.

### 2.4 Functional assay

Luciferase reporter assay was used to assess the effects of rs11133399 on the expression of *CLOCK* gene. The 2250 bp double‐strand DNA located in the 5′‐UTR of the *CLOCK* gene carrying either A or G genotype of rs11133399 was cloned into the pGL3‐basic vector (Promega, Madison, WI, USA). Luciferase reporter assay was performed in SGC‐7901 and AGS cells using a dual‐luciferase kit (Promega) as previously described.^16^

### 2.5 Immunohistochemistry

Formalin‐fixed, paraffin‐embedded GC tissues from patients with different genotypes of SNPs rs11133399 were collected and their hematoxylin‐eosin slides were viewed by a pathologist. Four micrometer thick sections were cut from corresponding blocks containing representative tumor regions. Immunohistochemistry (IHC) was performed as previously described using a rabbit antibody against human CLOCK (1:50; Abcam, Cambridge, MA, USA).^19^ The intensity and extent of immunostaining were assessed under double‐blinded conditions as previously described.^19^

### 2.6 Statistical analysis

All statistical analyses were conducted using the IBM SPSS Statistics 19.0 software (Armonk, NY, USA), and two‐sided *P* < 0.05 was considered statistically significant. Multivariate Cox regression analysis was used to assess the impact of clinical parameters and SNPs on the prognosis of patients as indicated by hazard ratio (HR) and 95% confidence interval (CI). The main analyses were performed under three genetic models (dominant, additive, and recessive) and the best‐fitting model (with the smallest *P* value) was selected for the association analysis. Cumulative effect was evaluated by combing the number of unfavorable genotypes identified from the main effect analysis of individual SNPs. Kaplan‐Meier analysis and log‐rank test were used to assess the prognosis difference in patients with different genotypes. Higher order gene‐gene interactions were estimated using survival tree analysis by STREE program (http://c2s2.yale.edu/software/stree/), which uses recursive partitioning to identify subgroups of individuals at higher risk. A receiver operating characteristic (ROC) curve was calculated to evaluate the specificity and sensitivity of prognostic prediction by different combinations of clinical and CPFL‐related genetic variables.

## 3 RESULTS

### 3.1 Patient characteristics and prognosis analysis

As shown in Table 1, 390 patients died of GC and 484 developed recurrence during the median of follow‐up of 58 months (range, 3‐112 months). Multivariate Cox regression analysis showed that late TNM stage, diffuse type, and poor differentiation were significantly associated with both poor RFS and OS in GC patients. In addition, adjuvant chemotherapy showed a significant protective effect on the prognosis of GC patients.

**Table 1:** Selected characteristics of GC patients and prognosis analysis

Characteristics	No. (%) n = 704	OS			RFS		
		Death (%) (n = 390)	HR^a^ (95% CI)	*P* value	Recurrence (%) (n = 484)	HR^a^ (95% CI)	*P* value
Age, years							
<58	339 (48.2)	182 (46.7)	Ref.		226 (46.7)	Ref.	
≥58	365 (51.8)	208 (53.3)	1.03 (0.84‐1.26)	0.763	258 (53.3)	1.07 (0.89‐1.28)	0.482
Sex							
Male	544 (77.3)	311 (79.7)	Ref.		384 (79.3)	Ref.	
Female	160 (22.7)	79 (20.3)	1.26 (0.97‐1.62)	0.079	100 (20.7)	**1.27 (1.02‐1.60)**	**0.035**
Tumor site							
Proximal	196 (27.8)	115 (29.5)	Ref.		136 (28.1)	Ref.	
Middle	257 (36.5)	140 (35.9)	0.95 (0.77‐1.28)	0.968	173 (35.7)	1.05 (0.84‐1.33)	0.659
Distal	251 (35.7)	135 (34.6)	0.93 (0.71‐1.24)	0.959	175 (36.2)	1.16 (0.93‐1.46)	0.196
TNM stage							
I	149 (21.2)	63 (16.2)	Ref.		78 (16.1)	Ref.	
II	343 (45.9)	189 (48.5)	**1.47 (1.08‐2.01)**	**0.014**	235 (48.6)	**1.57 (1.19 ‐ 2.08)**	**0.001**
III	157 (22.3)	97 (24.5)	**1.82 (1.28‐2.58)**	**0.001**	124 (25.6)	**2.11 (1.54 ‐ 2.88)**	**<0.001**
IV	55 (10.6)	41 (10.8)	**2.27 (1.49‐3.43)**	**<0.001**	47 (9.7)	**2.38 (1.62 ‐ 3.48)**	**<0.001**
Differentiation^b^							
Well	90 (12.8)	42 (15.8)	Ref.		55 (11.6)	Ref.	
Moderate	264 (37.5)	121 (40.4)	1.04 (0.75‐1.43)	0.811	160 (33.8)	1.12 (0.84‐1.50)	0.438
Poor	336 (47.7)	219 (43.8)	**1.39 (1.03‐1.78)**	**0.036**	259 (54.6)	**1.34 (1.02‐1.67)**	**0.042**
Unknown	14 (2.0)						
Lauren classification^b^							
Intestinal	293 (41.6)	135 (35.6)	Ref.		169 (36.0)	Ref.	
Diffuse	391 (55.5)	244 (64.4)	**1.55 (1.08‐1.89)**	**0.016**	301 (64.0)	**1.59 (1.12‐1.82)**	**0.007**
Other	20 (2.9)						
Chemotherapy^c^							
No	90 (18.0)	66 (23.1)	Ref.		73 (20.3)	Ref.	
Yes	410 (82.0)	220 (76.9)	**0.58 (0.44‐0.86)**	**0.009**	286 (79.7)	**0.65 (0.50‐0.84)**	**0.003**

The significant *P* values (≤0.05) were in bold.

TNM, tumor‐node‐metastasis; OS, overall survival; RFS, recurrence‐free survival; HR, hazard ratio; CI, confidence interval; Ref., reference; GC, gastric cancer.

a Adjusted for age, sex, tumor site, tumor stage, differentiation, Lauren classification, and chemotherapy where appropriate.

b Unknown differentiation and other classification were censored for further prognosis analysis due to the small number of subjects in these subgroups.

c Only including stage II and stage III GC patients received adjuvant chemotherapy for further prognosis analysis.

### 3.2 Association of single SNPs with clinical outcomes of GC patients

**Figure 1:**
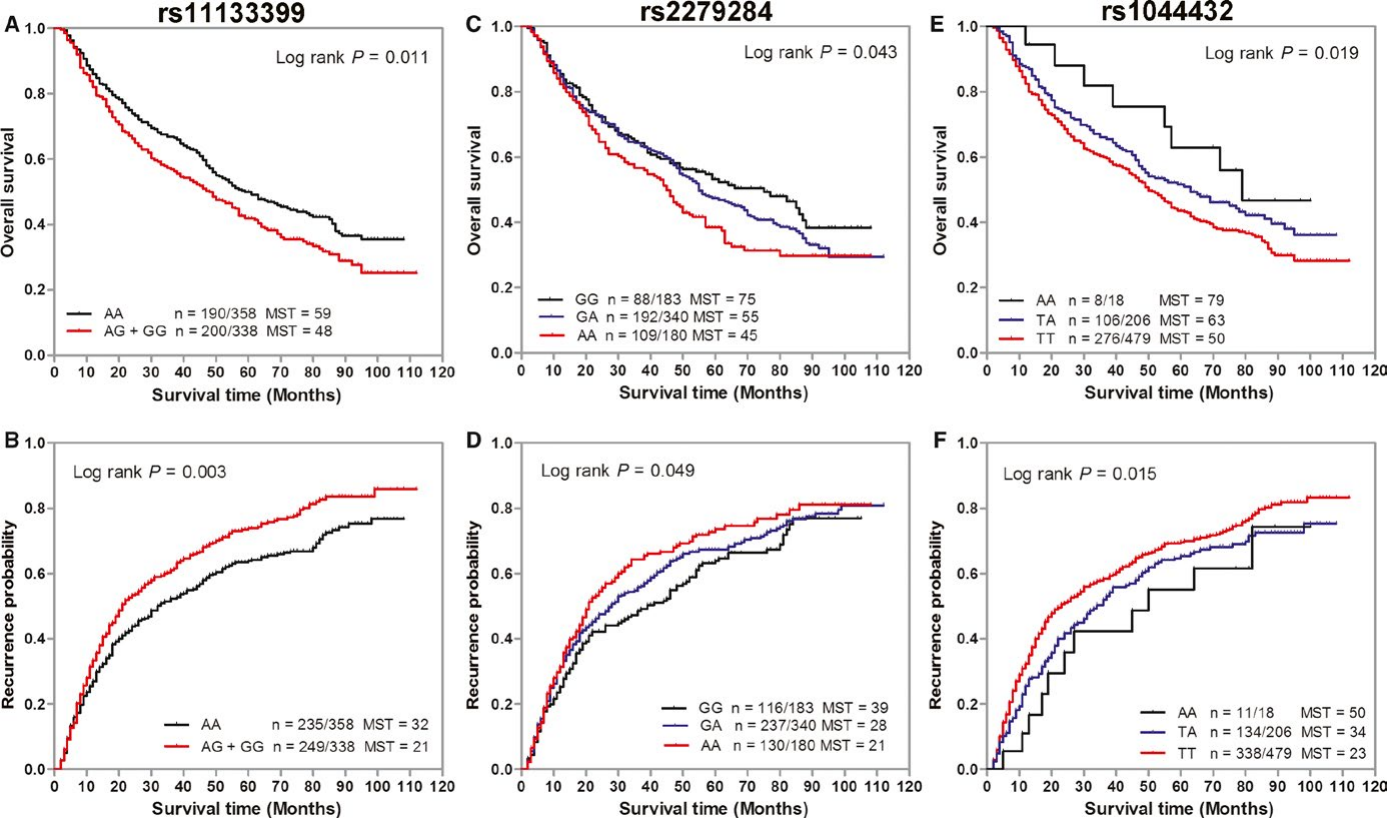
Kaplan‐Meier curves of gastric cancer (GC) patients stratified by genetic variants of circadian positive feedback loop genes. Overall survival and recurrence‐free survival of GC patients stratified by rs11133399 (A,B), rs2279284 (C,D), and rs1044432 (E,F). MST, median survival time

We evaluated the associations of each individual SNP with GC prognosis under dominant, additive, and recessive models, then presented the results with best‐fitting model (Table 2). Univariate Cox regression analysis showed that three SNPs had significant associations with the OS and RFS of GC patients. Among them, CLOCK rs11133399 was significantly associated with shorter GC OS or RFS under dominant model, with HRs of 1.29 (95% CI: 1.06‐1.57, *P* = 0.012) or 1.31 (95% CI: 1.10‐1.57, *P* = 0.003). Similar significant associations were observed between BAML1 rs2279284 and shorter GC OS or RFS under additive model, with HRs of 1.43 (95% CI: 1.08‐1.89, *P* = 0.013) or 1.14 (95% CI: 1.01‐1.29, *P* = 0.038). However, BAML1 rs1044432 had significant protective effect on GC OS or RFS under additive model, with HRs of 0.79 (95% CI: 0.65‐0.96, *P* = 0.019) and 0.81 (95% CI: 0.68‐0.96, *P* = 0.015). We further conducted a multivariate Cox regression analysis by adjusting for age, sex, tumor site, tumor stage, differentiation, Lauren classification, and chemotherapy. In our multivariable analysis, there were still significant associations of GC prognosis with CLOCK rs11133399 (for OS: HR 1.27, 95% CI: 1.04‐1.55, *P* = 0.020; for RFS: HR 1.28, 95% CI: 1.07‐1.54, *P* = 0.007), BAML1 rs2279284 (for OS: HR 1.18, 95% CI: 1.02‐1.36, *P* = 0.024; for RFS: HR 1.12, 95% CI: 1.01‐1.26, *P* = 0.048), and BAML1 rs1044432 (for OS: HR 0.82, 95% CI: 0.68‐0.99, *P* = 0.047; for RFS: HR 0.84, 95% CI: 0.71‐0.98, *P* = 0.042). Kaplan‐Meier analysis revealed that patients with rs11133399 AG/GG or rs2279284 GA/AA genotypes had worse OS and RFS than those with AA or GG genotype, while patients carrying rs1044432 TA/AA genotypes had better OS and RFS than those carrying TT genotype (Figure 1).

**Table 2:** Association of single SNPs in circadian positive feedback loop genes with clinical outcomes of GC patients

Gene	SNP	Predicted function	Best fitting model	OS				RFS			
				Crude HR (95% CI)	*P*	Adjusted HR^a^ (95% CI)	*P*	Crude HR (95% CI)	*P*	Adjusted HR^a^ (95% CI)	*P*
*CLOCK*	rs3749474	miRNA	Recessive	0.87 (0.64‐1.18)	0.358	0.82 (0.60‐1.12)	0.208	0.99 (0.76‐1.28)	0.915	0.92 (0.71‐1.20)	0.546
	rs1801260	miRNA	Additive	0.79 (0.60‐1.05)	0.105	0.81 (0.61‐1.07)	0.132	0.81 (0.64‐1.04)	0.093	0.83 (0.65‐1.06)	0.134
	rs11133399	TFBS	Dominant	**1.29 (1.06‐1.57)**	**0.012**	**1.27 (1.04‐1.55)**	**0.020**	**1.31 (1.10‐1.57)**	**0.003**	**1.28 (1.07‐1.54)**	**0.007**
*BMAL1*	rs2279284	TFBS	Additive	**1.43 (1.08‐1.89)**	**0.013**	**1.18 (1.02‐1.36)**	**0.024**	**1.14 (1.01‐1.29)**	**0.038**	**1.12 (1.00‐1.26)**	**0.049**
	rs1044432	miRNA	Additive	**0.79 (0.65‐0.96)**	**0.019**	**0.82 (0.68‐0.99)**	**0.047**	**0.81 (0.68‐0.96)**	**0.015**	**0.84 (0.71‐0.98)**	**0.042**
*NPAS2*	rs1562313	Splicing	Recessive	0.83 (0.53‐1.30)	0.415	0.85 (0.54‐1.34)	0.490	0.93 (0.64‐1.40)	0.812	0.95 (0.65‐1.41)	0.814
	rs9223	Splicing	Dominant	1.14 (0.93‐1.39)	0.205	1.10 (0.90‐1.34)	0.365	1.16 (0.97‐1.39)	0.103	1.10 (0.92‐1.32)	0.291
	rs2305158	miRNA	Dominant	0.82 (0.66‐1.02)	0.069	0.85 (0.68‐1.05)	0.136	0.84 (0.49‐1.01)	0.069	0.88 (0.72‐1.06)	0.174
	rs1053096	miRNA	Recessive	0.85 (0.65‐1.09)	0.199	0.85 (0.66‐1.10)	0.224	0.89 (0.71‐1.11)	0.289	0.89 (0.71‐1.12)	0.319

The significant *P* values (≤0.05) were in bold.

OS, overall survival; RFS, recurrence‐free survival; HR, hazard ratio; CI, confidence interval; GC, gastric cancer; SNP, single‐nucleotide polymorphism.

a Adjusted by age, sex, tumor site, tumor stage, differentiation, Lauren classification, and chemotherapy where appropriate.

### 3.3 Cumulative effects of unfavorable genotypes on the prognosis of GC patients

**Figure 2:**
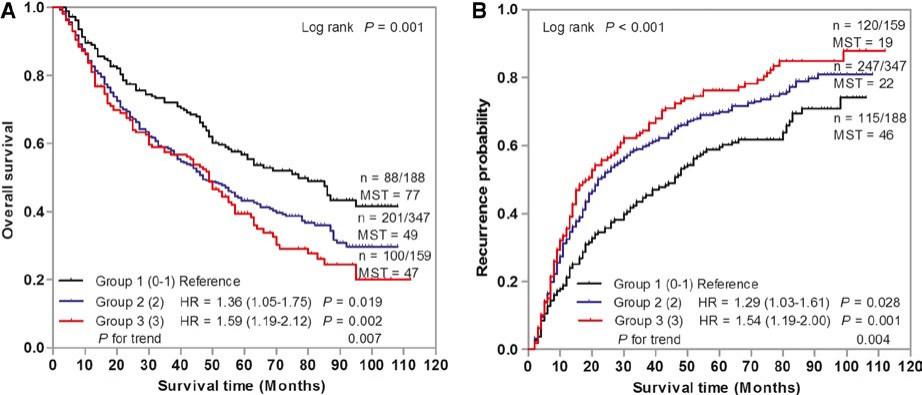
Cumulative analysis of unfavorable genotypes overall survival (A) and recurrence‐free survival (B) for gastric cancer patients. Hazard ratios (HRs) were adjusted for age, sex, tumor site, differentiation, Lauren classification, tumor‐node‐metastasis stage, and adjuvant chemotherapy. MST, median survival time

To assess the cumulative effects of multiple SNPs on GC prognosis, we combined the unfavorable genotype of each individual SNP and analyzed their associations with OS and RFS. As shown in Figure 2, both the risks of death and recurrence were elevated with the increasing of the number of unfavorable genotypes (*P* = 0.007 and 0.004, respectively). Kaplan‐Meier curves showed that both OS and RFS were significantly different among patients stratified with different number of unfavorable genotypes.

### 3.4 Prognosis prediction sensitivity of CPFL genotype combined with clinical parameters

**Figure 3:**
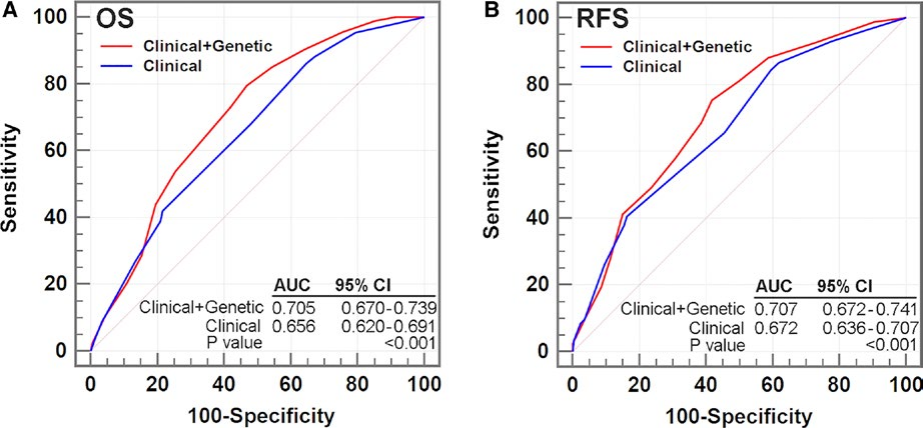
The prognostic performance of the combination of single‐nucleotide polymorphisms in circadian positive feedback loop genes with the clinical variables. Receiver operating characteristic analysis suggested that combined genetic and clinical variables model had a significant improvement of assessment ability than did clinical variables alone model in both (A) overall survival (OS) and (B) recurrence‐free survival (RFS). Clinical variables include age, sex, tumor site, differentiation, Lauren classification, and tumor‐node‐metastasis stage. Genetic variables include rs11133399, rs2279284, and rs1044432

Considering the prognostic predicting value of CPFL SNPs and the heterogeneity of GC prognosis, we assessed whether the combination of CPFL SNPs and clinical parameters would improve survival prediction. Area under the ROC curve (AUC) was calculated after sequentially adding clinical prognostic factors and the three SNPs (rs11133399, rs2279284, and rs1044432). As shown in Figure 3A, the AUC increased from 0.656 (clinical variables) to 0.705 (clinical variables plus three SNPs) for OS prediction. Similarly, the AUC increased from 0.672 (clinical variables) to 0.707 (clinical variables plus three SNPs) for RFS prediction (Figure 3B). These data suggested that addition of genetic variables of CPFL genes to clinical variables would improve GC outcome prediction.

### 3.5 Higher order gene‐gene interactions GC prognosis

**Figure 4:**
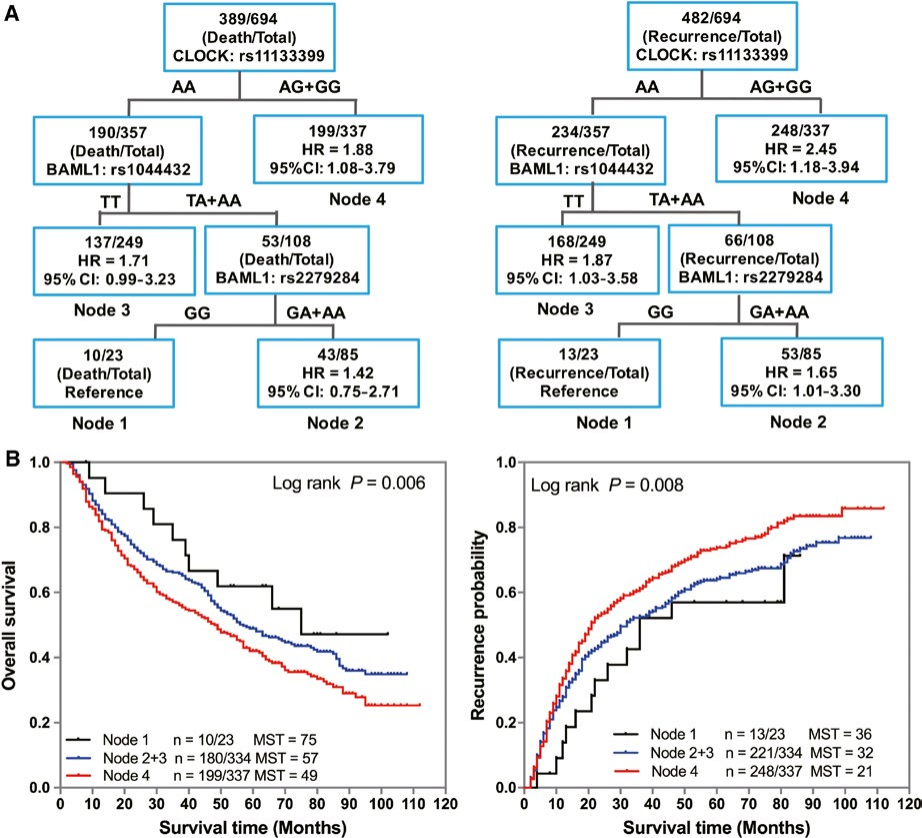
Potential higher order gene‐gene interactions among circadian positive feedback loop gene polymorphisms. Tree structure identifying subgroups of patients with different genetic backgrounds in overall survival (OS) and recurrence‐free survival (RFS) (A). Kaplan‐Meier survival curves for patients based on survival tree analysis in OS and RFS (B). Hazard ratios (HRs) were adjusted for age, sex, tumor site, differentiation, Lauren classification, tumor‐node‐metastasis stage, and adjuvant chemotherapy. MST, median survival time

To determine whether complex interactions among these SNPs would potentially affect GC patient prognosis, we performed survival tree analysis to assess the higher order gene‐gene interactions. As shown in Figure 4A, three SNPs exhibited gene‐gene interactions, leading to four terminal nodes with different OS or RFS. The initial split on the survival tree was due to CLOCK rs11133399 (Node 2), indicating that this SNP was the primary factor contributing to both OS and RFS differences in GC patients. The longest RFS was observed in patients of Node 1 group, which was composed of individuals with rs11133399 AA genotype, rs2279284 GG genotype, and rs1044432 TA/AA genotypes. The shortest RFS was observed in Node 4 group patients with rs11133399 AG/GG genotypes. Kaplan‐Meier curves significantly distinguished the survival of patients stratified by survival tree nodes (Figure 4B).

### 3.6 Functional effects of *CLOCK* rs11133399 on promoter activity and gene expression

**Figure 5:**
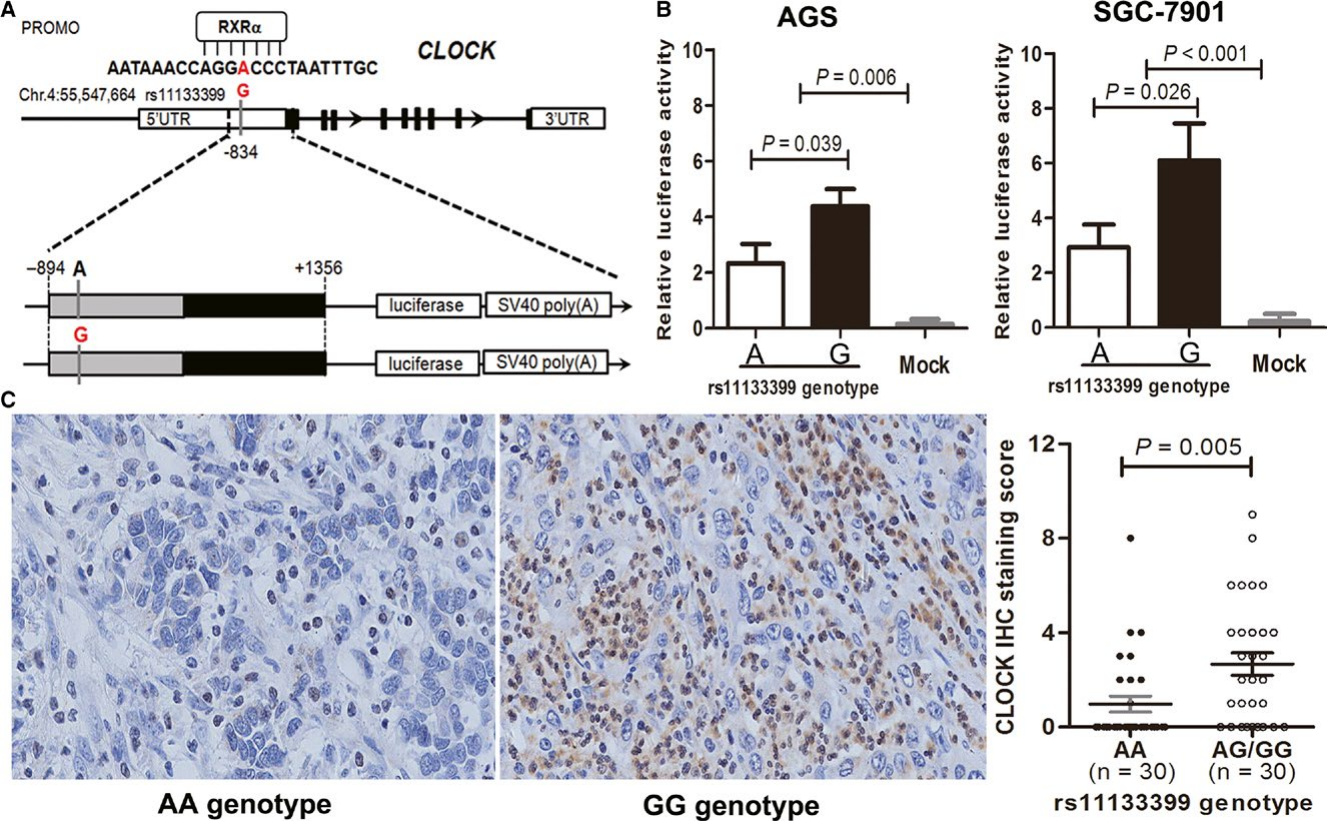
Effects of rs11133399 genotypes on the transcriptional activities and expression levels of CLOCK in GC. (A) Schematic representation of the human *CLOCK* gene. Schematic representation is shown according to GRCh38.p7 assembly. Arrows indicate direction of transcription. Black boxes on the arrow line represent exons. SNP rs11133399 is located within RXRα binding region at the 5′‐UTR of *CLOCK*. The two types of promoter reporter constructs are shown below the *CLOCK* gene, each with the major (black) and minor/risk (red) allele listed. (B) Comparison of luciferase activities in SGC‐7901 and AGS cells transfected with promoter reporter constructs containing rs11133399 A allele (pGL3‐CLOCK‐A) or G allele (pGL3‐CLOCK‐C). (C) Representative picture of immunohistochemical staining for CLOCK in GC tissues with rs11133399 AA or GG genotype. Magnification, ×200

We further employed the ALGGEN PROMO 3.0 software (http://alggen.lsi.upc.edu/recerca/menu_recerca.html) to explore the potential biological effects of rs11133399 and found that this SNP maps within a canonical RXRα‐binding site at the 5′‐UTR of *CLOCK* gene (Figure 5A), indicating that rs11133399 might affect the transcription of its downstream gene. To test this hypothesis, SGC‐7901 and AGS GC cells were transfected with luciferase reporter plasmid constructs containing the 5′‐UTR of *CLOCK* gene with either rs11133399 A or G genotype. Our results showed that rs11133399 genotype significantly influenced the normalized luciferase activity in all transfected cells. Cells transfected with plasmid construct carrying G on rs11133399 exhibited significant increased normalized luciferase activity than those transfected with A genotype on rs11133399 (Figure 5B). We further investigated the expression of CLOCK in 60 GC tissues with different rs11133399 genotypes (30 with AG/GG genotypes and 30 with AA genotype) using IHC. As shown in Figure 5C, CLOCK protein level was significantly higher in patients with AG/GG genotypes than in those with AA genotype (*P* = 0.005).

## 4 DISCUSSION

The prognosis of GC survival has been reported to be predicted by many factors. Cheong et al. have reported that single patient classifiers (based on the expression of GZMB, WARS, and SFRP4) provide clinically important prognostic information independent of standard risk‐stratification methods and predicted chemotherapy response after surgery in two independent cohorts of patients with resectable, stage II‐III GC.^20^ Neutrophils are enriched predominantly in the invasive margin of GC tissues and increased neutrophil counts in the peripheral blood are significantly associated with poor prognosis in GC patients.^21^ The multivariate analysis has revealed that a GC‐support vector machine prognostic classifier is an independent prognostic factor.^22^ The classifier had higher predictive accuracy for OS and disease‐free survival than TNM stage and can complement the prognostic value of the TNM staging system. These findings provide different methods to predict survival for GC patients. In this study, we evaluated the effects of nine functional SNPs in the three CPFL genes (*CLOCK*,* NPAS2*, and *BMAL1*) on the prognosis of a cohort of Chinese GC patients. We found that three SNPs (rs11133399 in *CLOCK*, rs1044432, and rs2279284 in *BAML1*) were significantly associated with both OS and RFS of GC patients. Additionally, we observed an accumulative risk of death and relapse with the increasing number of unfavorable genotypes and combination of CPFL genotype and clinical factors significantly improved prognosis prediction of GC patients. Survival tree analysis revealed that SNP rs11133399 in *CLOCK* gene was the primary factors contributing to both OS and RFS of GC patients. Moreover, our functional assay indicated that rs11133399 had a significant impact on the expression of *CLOCK* in both GC cell lines and tissues. These data collectively suggest that polymorphisms in CPFL genes may be a useful predicting factor for GC prognosis.

It has long been proposed that disruption of circadian rhythm may contribute to the development of cancer,^2^ and shift work involving circadian disruption has been classified as a probable carcinogen to human beings by the International Agency for Research on Cancer.^23^ Epidemiological studies have shown that circadian disruption is significantly associated with increased risk of a range of malignancies, such as breast and prostate cancer.^3^, ^4^ As important transcription factors, circadian genes play important roles in the regulation of gene expression, including those that are involved in DNA damage repair, cell proliferation, apoptosis, and cell cycle control.^24^, ^25^ Recent studies have revealed that dysregulation of circadian genes is involved in the development of cancer in both humans and rodents,^26^ and the expression level of circadian genes is associated with the prognosis and chemotherapy sensitivity.^27^, ^28^ Therefore, elucidating the biological roles of circadian genes in the procedure of carcinogenesis will be helpful for cancer prevention and treatment.

The molecular mechanisms of circadian clock genes in the occurrence and development of tumors remain unclear. It has reported that clock genes contribute to the occurrence and development of tumors by regulating and interfering with clock controlled genes, such as oncogenes (c‐myc), tumor suppressor genes (p53 and p21), genes involved in the regulation of the cell cycle (cyclins A, B1 and D1, and WEE1 G2 checkpoint kinase), and vascular endothelial growth factor as well as affecting the internal secretion pathway.^29^, ^30^, ^31^ These target genes regulated by the biological clock genes are involved in DNA damage repair, cell proliferation, and apoptosis.^29^ Therefore, circadian clock disorders may lead to uncontrolled cell growth and malignant transformation. However, the exact mechanisms of abnormal expression of clock genes in tumors and their functional role in tumor occurrence and progression need further investigations.

Genetic variants such as SNPs play an important role in the regulation of gene expression, mRNA translation and degradation, and protein structures, all of which may affect gene functions and human phenotype.^32^ Considering the important biological roles of circadian genes in cancer development, it is reasonable that SNPs in these genes may affect cancer cell proliferation, invasion, and treatment sensitivity, and thus affect cancer susceptibility and patient outcome. Molecular epidemiological studies have demonstrated that polymorphisms in circadian genes are associated with the risk of a number of types of cancer, such as breast, ovarian, prostate cancer, and non‐Hodgkin lymphoma.^10^, ^11^, ^33^, ^34^, ^35^, ^36^ CLOCK‐BMAL1 heterodimer is at the heart of the molecular circadian autoregulatory feedback loop. Previous studies have suggested that polymorphisms in either *BAML1* or CLOCK are associated with several types of cancer.^14^, ^37^ Our previous findings have demonstrated that functional SNPs in *CLOCK* gene are significantly associated with prognosis of CRC.^38^ Yuan et al. have shown that *NPAS2* polymorphisms are associated with the death risk of HCC patients after transcatheter arterial chemoembolization treatment.^39^ In line with these findings, we found that rs11133399 in *CLOCK*, rs1044432 and rs2279284 in *BAML1* are significantly associated with the prognosis of GC patients. These data suggest that different SNPs in circadian genes might play different roles in the initiation and progression of different malignancies. However, the concrete biological functions of these SNPs in specific cancer types need further investigation.

Of particular concern, *CLOCK* rs11133399 was found to be associated with an increased risk of death and recurrence in GC patients. However, its underlying mechanisms remain unclear. Besides to its role in the circadian rhythm maintaining, CLOCK also directly or indirectly regulates a number of clock‐controlled genes with various biological functions, including those associated with carcinogenesis.^24^, ^40^ As a transcriptional enhancer, CLOCK can directly regulate genes important for cell cycle control, and *Clock* deficiency significantly suppresses cell proliferation and malignant transformation.^41^, ^42^ Hoffman et al. have found that CLOCK expression is elevated in human breast cancer tissues and is associated with a cancer‐relevant network of transcripts.^43^ Puram et al. have demonstrated that *Clock* and *Bmal1* are required for murine AML cell proliferation in vitro and in vivo.^44^ Circadian pathway disruption leads to impair of leukemic cell proliferation, enhancement of myeloid differentiation, and depletion of leukemia stem cells. In this study, our bioinformatic analysis showed that rs11133399 maps at the 5′‐UTR of *CLOCK* gene within a canonical RXRα‐binding site which would block *CLOCK* gene transcription after RXRα binding. Our subsequent functional assays revealed that G allele in rs11133399 could significantly enhance the transcriptional activity of *CLOCK* gene in GC cells. IHC staining further demonstrated that the deleterious G genotype of rs11133399 was associated with higher CLOCK protein expression in GC tissues, suggesting that polymorphisms of *CLOCK* might influence the biological aggressiveness of cancer by affecting gene expression and ultimately contribute to determine patient prognosis. However, the detailed molecular mechanisms by which these SNPs affect the transcriptional activity and expression of CLOCK need further investigation.

Nevertheless, there are opposite findings on the prognostic effects of *CLOCK* gene polymorphisms. Rajendran et al. have recently found that the increased number of *CLOCK* alleles linked to lower gene expression (ie, C of rs3749474 and G of rs1801260) is associated with poor prognosis of GC patients, although each individual SNP has no effect on the prognosis of patients.^15^ This discrepancy may stem from the complex association between circadian rhythm and cancer. Korkmaz et al. have recently observed opposite carcinogenic effects of BMAL1 in breast cancer.^45^ Moreover, genetic background between research populations may also contribute to this disagreement. Therefore, further studies are needed to comprehensively elucidate the geological roles of *CLOCK* polymorphisms in the development and progression of different types of cancer.

Another finding of this study was that rs2279284 and rs1044432 in *BMAL1* were closely related to GC prognosis. As an important partner of CLOCK, BMAL1 has been generally considered as a tumor suppressor in several types of cancer,^46^, ^47^ while several studies have suggest that BMAL1 has the potential to promote tumor growth and progression.^37^, ^44^ However, to date, no study has been focused on the biological roles of rs2279284 and rs1044432 in cancer. In silico analysis indicated that rs2279284 is located in the transcriptional factor binding sites, while rs1044432 within the microRNA binding region of *BMAL1*. Jiang et al have recently demonstrated that miR‐135b‐induced BMAL1 repression by direct 3′‐UTR targeting promotes pancreatic tumourigenesis and chemoresistance.^48^ Therefore, these two SNPs could influence *BMAL1* gene expression, mRNA stability, or protein function in GC cells and finally affect the aggressiveness of GC. These functional assumptions might underlie the molecular mechanisms by which GC prognosis is affected. However, further experimental studies are needed to test this hypothesis.

It is well known that cancer prognosis is significantly influenced by intricate interactions between host genetic factors and tumor characteristics.^1^ Since our findings linked CPFL SNPs with GC prognostic assessment, we incorporated the three significant SNPs into a multivariate outcome assessment model and found a significant improvement of discriminatory ability. In addition, we further explored the higher order gene‐gene interactions among CPFL SNPs and their association with patient prognosis using survival tree analysis. We found that *CLOCK* rs11133399 was the primary split in the survival tree that had the strongest impact on patient survival, indicating that this SNP may account more for GC development and progression.

Our study has several limitations. Firstly, we could not rule out the possibility of chance findings in our study due to the lack of external validation. In addition, our study was restricted to Han Chinese and whether the findings can be generalized to other ethnic groups needs further evaluations. Larger multiethnic and multicenter studies are warranted in the future.

In summary, our findings provide an insight that CPFL gene polymorphisms are significantly associated with the prognosis of GC patients. Functional studies are needed to investigate the underlying mechanisms to imply our results.

## CONFLICT OF INTEREST

The authors declare that they have no conflict of interest.
